# Optimal DL-Methionyl-DL-Methionine Supplementation Improved Intestinal Physical Barrier Function by Changing Antioxidant Capacity, Apoptosis and Tight Junction Proteins in the Intestine of Juvenile Grass Carp (*Ctenopharyngodon idella*)

**DOI:** 10.3390/antiox11091652

**Published:** 2022-08-25

**Authors:** Pei Wu, Yuening Su, Lin Feng, Weidan Jiang, Shengyao Kuang, Ling Tang, Jun Jiang, Yang Liu, Xiaoqiu Zhou

**Affiliations:** 1 Animal Nutrition Institute, Sichuan Agricultural University, Chengdu 611130, China; 2 Fish Nutrition and Safety Production University Key Laboratory of Sichuan Province, Sichuan Agricultural University, Chengdu 611130, China; 3 Key Laboratory of Animal Disease-Resistance Nutrition, Ministry of Education, Ministry of Agriculture and Rural Affairs, Key Laboratory of Sichuan Province, Chengdu 611130, China; 4 Animal Nutrition Institute, Sichuan Academy of Animal Science, Sichuan Animtech Feed Co., Ltd., Chengdu 610066, China; 5 College of Animal Science and Technology, Sichuan Agricultural University, Chengdu 611130, China

**Keywords:** methionyl-methionine, grass carp, intestinal physical barrier, Nrf2 signaling, caspases, claudins

## Abstract

The present study was a part of a larger research project that aimed to investigate the effects of Met-Met supplementation on fish growth and intestinal health. This study mainly focused on the relationship between dietary Met-Met and intestinal physical barrier function in fish. Seven iso-nitrogenous diets supplemented with 2.50 g/kg DL-methionine (DL-Met) and six graded levels of Met-Met (0.00, 0.79, 1.44, 1.84, 2.22, and 2.85 g/kg) were used to feed juvenile grass carp for 10 weeks, after which a 14-day *Aeromonas hydrophila* challenge test was performed. The results indicated that optimum levels of Met-Met decreased intestinal oxidative damage, probably by increasing total antioxidant capacity, and the activity and gene expression levels of several antioxidant enzymes, which were closely related to the changed Nrf2/Keap1 signaling. Meanwhile, optimum levels of Met-Met decreased intestinal apoptosis and improved the intestinal tight junction, as evident by the downregulated mRNA levels of initiator and executioner caspases; the pro-apoptotic-related proteins *FasL*, *Apaf-1*, and *Bax*; and upregulated mRNA levels of the anti-apoptotic proteins *Bcl-2*, *Mcl-1b*, and *IAP* and the TJ proteins *claudins*, *occludin*, and *ZOs*. Furthermore, the positive effects of Met-Met on improving intestinal physical barrier function were superior to those of DL-Met in fish. These findings showed that optimal Met-Met supplementation improved intestinal physical barrier function, probably by changing antioxidant capacity, apoptosis, and tight junction proteins in fish.

## 1. Introduction

Because of its shortage and high cost, replacing fishmeal with plant proteins has been a major objective for sustainable aquaculture [1]. Moreover, several plant oil and extracts, such as pomegranate seed oil [2], hot pepper (*Capsicum* sp.) oil [3], and *Thymus vulgaris* essential oil [4], are usually beneficial for fish growth and health. However, using plant proteins to replace dietary fishmeal usually results in essential amino acid (EAA) deficiencies [5]. Adding crystalline amino acids (CAAs) to plant-ingredients-based diets is likely to become an increasingly common practice to optimize the nutritional value of these diets. It has been found that crystalline amino acid supplementation improved fish growth and protein synthesis, as well as immunity and antioxidant capacity [6,7]. Methionine is a major limiting EAA in most plant-based ingredients of fish feed [1]. Studies have shown that supplementing a plant-ingredients-based diet with DL-methionine improved the growth performance of rainbow trout (*Oncorhynchus mykiss*) [1] and juvenile cobia (*Rachycentron canadum*) [5]. However, CAAs was not utilized as efficiently as AAs in protein, partly because of the faster leaching of CAAs, as reported in young yellowtail (*Seriola quinqueradiata*) [8], juvenile *Cyprinus carpio* Var. Jian [9], and juvenile Chinese sucker (*Myxocyprinus asiaticus*) [10]. Consequently, decreasing the leaching rate of purified AAs would enhance their utilization. DL-methionyl-DL-methionine dipeptide (Met-Met) has low water solubility and high hydrothermal stability, and can be cleaved enzymatically by fish under physiological conditions to provide free D- and L-methionine, and thus it has been used as a methionine supplement for aquaculture to reduce the leaching losses of methionine [11].

Up to now, supplementation with Met-Met has improved the growth of juvenile grass carp (*Ctenopharyngodon idella*) [12], juvenile Nile tilapia (*Oreochromis niloticus*) [13], and white leg shrimp (*Litopenaeus vannamei*) [14,15]. Fish growth and health is closely correlated to intestinal barrier function, as the intestine is not only an important organ for nutrients absorption, but is also a vital barrier that could restrict the access of harmful agents [16]. The physical barrier and the immune barrier are vital parts of the intestinal barrier in fish. Our previous study indicated that optimal Met-Met addition enhanced intestinal immunity in juvenile grass carp [12]. However, little information about the relationship between Met-Met and the intestinal physical barrier is available for fish. The intestinal physical barrier is mainly made up by the enterocytes and tight junctions [16], and could be damaged by oxidative stress and abnormal apoptosis of the intestinal epithelial cells [17]. Limited studies have shown that optimal levels of Met-Met enhanced the total antioxidant capacity of Nile tilapia liver [13] and white leg shrimp plasma [15]. In mammal, Met-Met up-regulated Janus kinase 2 (JAK2) mRNA level in bovine mammary tissue explants [18]. JAK2 improved the mRNA levels of the anti-apoptosis protein B-cell lymphoma protein-2 (Bcl-2) and prevented cell death in murine hematopoietic cells [19]. Additionally, the expression of two tight junction proteins, claudin-1 and ZO-1, in the jejunum of squabs was increased by dietary Met-Met supplementation [20]. These observations revealed that optimal Met-Met addition might maintain the intestinal physical barrier in fish by modulating the antioxidant ability, cellular apoptosis, and intracellular tight junctions of the intestine, which is worth researching.

This study expands on our previous study, which found that optimal Met-Met addition enhanced fish growth performance and intestinal immunity [12], and aims to fill the knowledge gap regarding Met-Met and intestinal physical barrier function in fish by analyzing intestinal antioxidants, cellular apoptosis, and tight junction proteins in juvenile grass carp, which could provide more information revealing the mechanisms whereby Met-Met enhances fish intestinal health. Moreover, the optimum Met-Met supplementation for grass carp juveniles based on intestinal physical barrier function and a comparison of the effects of Met-Met and DL-Met are also evaluated to give a reference for formulating commercial feed for grass carp.

## 2. Materials and Methods

### 2.1. Diets, Animal and Experimental Design

The details of the experimental procedures were similar to those previously described by Su et al. [12]. In brief, a basal diet based on fishmeal, soybean meal, and cottonseed meal was formulated to satisfy the nutrient requirements, except for methionine, of juvenile grass carp. The composition of the basal diet is presented in Table 1. The contents of methionine and cysteine in the basal diet (methionine deficiency) were measured to be 4.26 and 3.77 g/kg, respectively, by high-performance liquid chromatography (HPLC), as described by Pan et al. [21]. The positive control diet was formulated to meet the fishes’ requirements for total sulfur amino acids by adding DL-methionine (DL-Met) at a level of 2.50 g/kg to the basal diet. The six Met-Met diets were formulated by supplementing 0.00 (negative control), 1.00, 1.50, 2.00, 2.50, and 3.00 g/kg Met-Met (Evonik, Essen, Germany) to the basal diet, and the Met-Met concentrations in the six diets were analyzed to be 0.00, 0.79, 1.44, 1.84, 2.22, and 2.85 g/kg according to the HPLC method. All the seven experimental diets were iso-nitrogenous and iso-lipidic.

After being obtained from a local fishery (Sichuan, China), healthy juvenile grass carp were raised within net cages (1.5 m × 0.8 m × 1.5 m) for 4 weeks of acclimatization. Next, 2520 fish (10.10 ± 0.03 g body weight) in total were allocated to 7 groups randomly with 6 replicates per group (42 cages in total, 60 fish in each cage), and fed to apparent satiation with the respective experimental diets four times every day (i.e., 7:00, 11:00, 15:00, and 19:00) for 70 days. During the feeding trial, dissolved oxygen, water temperature, and pH values were ≥6.0 mg/L, 27 ± 2 °C, and 7.0 ± 0.4, respectively, and the light conditions followed a natural light/dark cycle (approximately 13/11 h).

### 2.2. Challenge Test and Sampling

A detailed description of the challenge test has been previously reported [12]. In brief, after the growth trial, 60 fish were chosen from each group and inoculated intraperitoneally with a 1.0 mL nonlethal dosage of *A. hydrophila* (2.5 × 10^6^ CFU/mL). During the 14-day infection period, the same diets as in the feeding trial were fed to the grass carp. At the end of the challenge test, all grass carp in each group were anaesthetized using a benzocaine bath (50 mg/L) and sacrificed by a blow on the head, and the intestines were removed, divided into three parts (proximal intestine (PI), mid intestine (MI), and distal intestine (DI)), then immediately frozen in liquid nitrogen, followed by storage at −80 °C. The degree of intestinal red coloration, swelling, and hyperemia of the grass carp was recorded in our previous study [12].

### 2.3. Biochemical Analysis

For biochemical analysis, six intestines from each group were homogenized following the kit instructions. The contents of protein carbonyl (PC), malondialdehyde (MDA), and reactive oxygen species (ROS); total antioxidant capacity (T-AOC), anti-hydroxyl radical (AHR), and anti-superoxide anion (ASA) capacity; activity of antioxidant enzymes (i.e., superoxide dismutase (SOD), glutathione peroxidase (GPx), glutathione-S-transferase (GST), catalase (CAT), and glutathione reductase (GR)); and the content of reduced glutathione (GSH) were analyzed with commercial kits from Nanjing Jiancheng Bioengineering Institute (Nanjing, China) in line with the specific protocols.

### 2.4. mRNA Abundance Analysis

Following the manufacturer’s instruction, the intestinal total RNA was obtained using the RNAiso Plus kit (TaKaRa, Dalian, China). Spectrophotometric (A260/A280) analysis and 1% agarose gel electrophoresis were used to determine the RNA quantity and quality. cDNA was obtained by reverse transcription using the PrimeScript™ RT reagent kit (TaKaRa, Dalian, China). Subsequently, quantitative real-time PCR was conducted using a commercial kit (SYBR Green, Takara, Dalian, China) in a real-time PCR detection system (CFX96, Bio-Rad, Hercules, CA, USA). The sequences of the specific primers are shown in Table 2. On the basis of our preliminary experiment results, β-actin was used as a reference gene (data not shown). Relative mRNA levels of the target genes were calculated by the 2^−ΔΔCT^ method after verifying the amplification efficiency of the primers to be approximately 100%.

### 2.5. Statistical Analysis

All data were presented as means and SD, and statistically analyzed using SPSS software 27.0 (SPSS Inc., Chicago, IL, USA). After the normality and homoscedasticity assumptions has been confirmed, one-way ANOVA and Duncan’s multiple range test were used to assess the differences among groups at *p* < 0.05. GraphPad Prism 8.0 (GraphPad Inc., La Jolla, CA, USA) was used for data visualization.

## 3. Results

### 3.1. Effects of Supplemented Met-Met on Antioxidant-Related Parameters in Grass Carp Intestines

**Figure 1:**
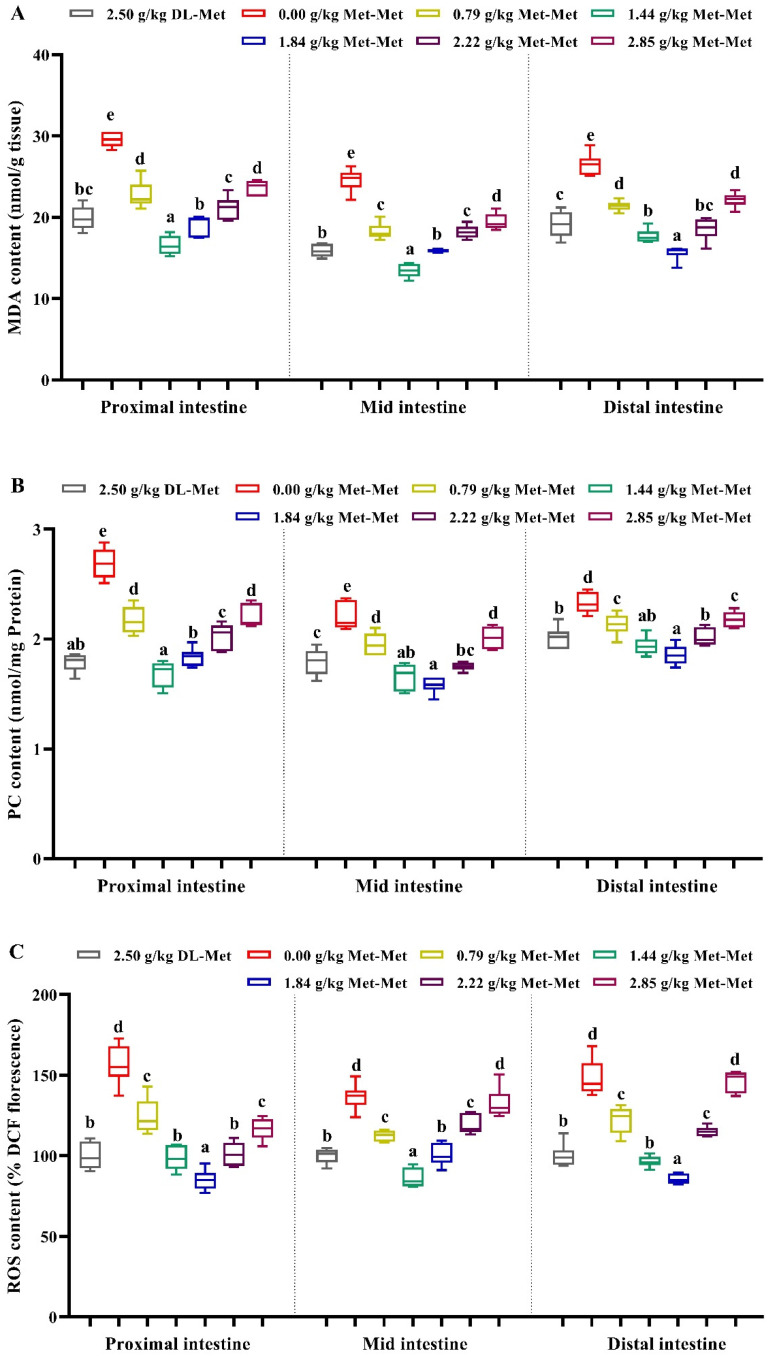
Effects of dietary DL-Met and Met-Met supplementation on malondialdehyde (MDA, (**A**)), protein carbonyl (PC, (**B**)), and reactive oxygen species (ROS, (**C**)) contents in three intestinal segments of juvenile grass carp. Data represent means of six fish in each group, error bars indicate S.D. ^a, b, c, d, e^ within a column, means without a common lowercase superscript differ (*p* < 0.05).

**Figure 2:**
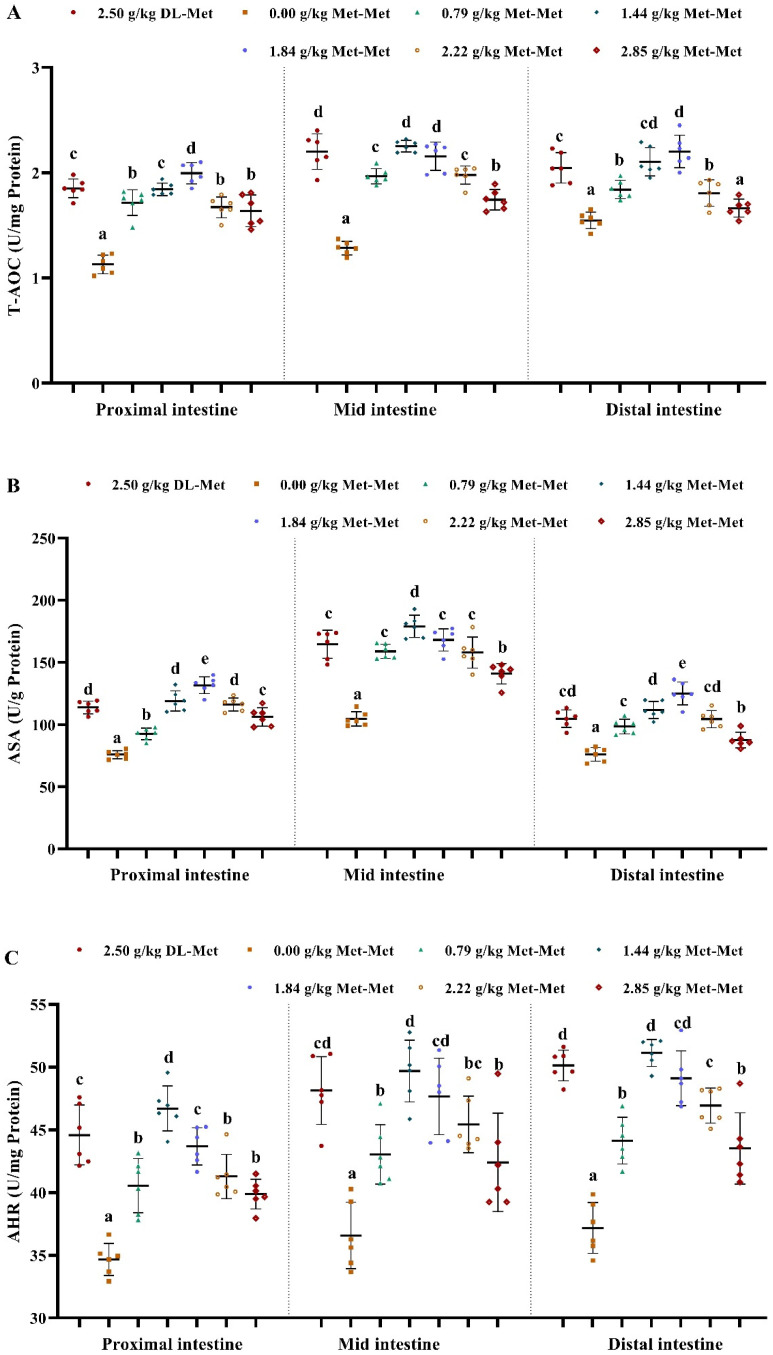
Effects of dietary DL-Met and Met-Met supplementation on total antioxidant capacity (T-AOC, (**A**)), anti-superoxide anion activity (ASA, (**B**)), and anti-hydroxyl radical activity (AHR, (**C**)) in three intestinal segments of juvenile grass carp. Data represent means of six fish in each group, error bars indicate S.D. ^a, b, c, d, e^ within a column, means without a common lowercase superscript differ (*p* < 0.05).

As presented in Figure 1, compared with the negative control group (Met deficiency, 0.00 g/kg Met-Met), the positive control group (group supplemented with 2.50 g/kg DL-Met) and the Met-Met supplementation groups significantly reduced the MDA, PC, and ROS content in the three intestinal segments of juvenile grass carp (*p* < 0.05). However, compared with the optimal Met-Met groups (1.44 or 1.84 g/kg), higher levels of Met-Met significantly increased the contents of MDA, PC, and ROS in fish intestines (*p* < 0.05). Contrary to the changes in MDA, the activity of T-AOC, ASA, and AHR in the PI, MI, and DI significantly increased with an increase in the supplemented Met-Met levels up to 1.44 or 1.84 g/kg (*p* < 0.05) (Figure 2). Furthermore, intestinal T-AOC and the activity of ASA and AHR in the positive control group were higher than those in the negative control group (the Met-deficient group) (*p* < 0.05) (Figure 2).

In the PI, the activity of T-SOD, MnSOD, and GR in the 1.84 g/kg Met-Met group and GST activity and GSH content in the 1.44 g/kg Met-Met group were significantly higher than those in other Met-Met groups (*p* < 0.05), whereas the activity of CAT and GPx was enhanced as Met-Met supplementation increased to 1.44 g/kg (*p* < 0.05) and then plateaued (Table 3). In the MI, the activity of T-SOD, MnSOD, and GPx in the 1.84 g/kg Met-Met group; the activity of GST and GR in the 1.44 g/kg Met-Met group; and the GSH content in groups with 1.44 and 1.84 g/kg Met-Met were higher than those in the other Met-Met-supplemented groups (*p* < 0.05), whereas CAT activity was increased in response to an increase in Met-Met up to 0.79 g/kg (*p* < 0.05), followed by a plateau (Table 3). In the DI, the activity of T-SOD, MnSOD, and GR, as well as GSH content in the group with 1.84 g/kg Met-Met; CAT and GPx activity in the group with 1.44 g/kg Met-Met; and GST activity in the 1.44 and 1.84 g/kg Met-Met groups were higher than those in the other Met-Met-supplemented groups (*p* < 0.05) (Table 3). However, supplemented DL-Met and Met-Met had no significant effect on intestinal CuZnSOD activity (*p* > 0.05).

As shown in Table 4, in comparison with the negative control group (the Met-deficient group), the relative gene expression levels of *MnSOD*, *GPx1a*, *GSTO1*, *GSTO2*, and *GR* were significantly upregulated by optimal Met-Met supplementation to 1.84 g/kg (*p* < 0.05), and *CAT*, *GPx4a*, *GPx4b*, *GSTP2*, and *GSTR* were significantly upregulated by Met-Met supplementation to 1.44 g/kg (*p* < 0.05); after that, the expression levels of these genes showed a decrease with a further enhancement in Met-Met; however, *GPx1b* was upregulated significantly by optimal Met-Met supplementation to 0.79 g/kg (*p* < 0.05), followed by a plateau. As presented in Table 5, compared with the negative control group (the Met-deficient group), the relative gene expression levels of *MnSOD*, *GPx4a*, *GSTP2*, and *GR* were significantly upregulated with increased Met-Met levels up to 1.44 g/kg (*p* < 0.05), while *GPx1a*, *GPx1b*, *GPx4b*, *GSTO1*, *GSTO2*, and *GSTR* mRNA levels were significantly upregulated with Met-Met supplementation to 1.84 g/kg (*p* < 0.05) and *CAT* was upregulated significantly by Met-Met supplementation to 0.79 g/kg (*p* < 0.05), followed by a plateau. As presented in Table 6, the relative gene expression levels of antioxidant enzymes in the DI showed similar patterns to those in the PI and MI. Furthermore, the relative gene expression levels of *MnSOD*, *GPx1a*, *GPx1b*, *GPx4a*, *GPx4b*, *GSTO1*, *GSTP2*, *GSTR*, and *GR* in the PI, MI, and DI in the negative control group (the Met-deficient group) were significantly lower than those in the positive control (DL-Met-supplemented group). However, the relative gene expression levels of *CuZnSOD* and *GSTP1* in the PI, MI, and DI were not significantly influenced by Met-Met supplementation (*p* > 0.05).

Furthermore, mRNA levels of the key regulatory molecule *Nrf2* in the PI, MI, and DI were significantly upregulated by the enhancement of Met-Met levels to 1.44 g/kg (*p* < 0.05), but after that, they declined with further increase in Met-Met (Table 4, Table 5 and Table 6). Conversely, the relative expression levels of *Keap1a* and *Keap1b* in the PI, MI, and DI were significantly downregulated with Met-Met supplementation to 1.44 g/kg (*p* < 0.05) and increased with a further increase in Met-Met (Table 4, Table 5 and Table 6). 

### 3.2. Effects of Supplemented Met-Met on Apoptosis-Related Parameters in Grass Carp Intestine

**Figure 3:**
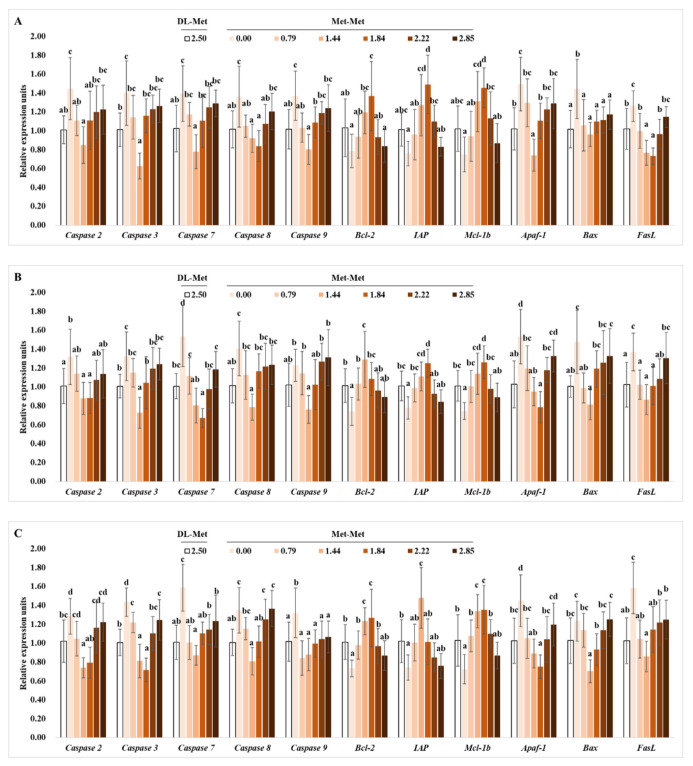
Effects of dietary DL-Met and Met-Met supplementation (g/kg diet) on relative expressions of apoptotic-related genes in the proximal intestine (**A**), mid intestine (**B**), and distal intestine (**C**) of juvenile grass carp. Data represent means of six fish in each group, error bars indicate S.D. ^a, b, c, d^ within a column, means without a common lowercase superscript differ (*p* < 0.05).

As shown in Figure 3A, in the PI, the relative gene expressions of cysteinyl aspartic acid-protease 2 (caspase-2), -3, -7, -8, -9, fatty acid synthetase ligand (FasL), and apoptotic protease activating factor-1 (Apaf-1) were significantly downregulated with Met-Met supplementation at levels of 1.44 or 1.84 g/kg in the diet (*p* < 0.05). Meanwhile, the relative gene expression levels of Bcl-2-associated X protein (Bax) were significantly downregulated with dietary Met-Met levels up to 0.79 g/kg (*p* < 0.05), followed by a plateau (*p* > 0.05). However, the relative gene expression levels of B-cell lymphoma protein-2 (Bcl-2), myeloid cell leukemia-1b (Mcl-1) and inhibitor of apoptosis proteins (IAP) were upregulated significantly by Met-Met supplementation up to 1.84 g/kg (*p* < 0.05). The expression levels of these genes in the positive control group were not different from those in the group supplemented with 0.79 g/kg Met-Met. 

The relative gene expression levels of *caspase-2*, *-3*, *-7, -8*, *-9*, *FasL*, *Apaf-1*, and *Bax* in the MI were significantly downregulated with increased Met-Met up to 1.44 or 1.84 g/kg (*p* < 0.05), respectively (Figure 3B). Meanwhile, the relative gene expression levels of *Bcl-2*, *Mcl-1*, and *IAP* in the MI were upregulated significantly with Met-Met supplementation to 1.44 or 1.84 g/kg (*p* < 0.05), respectively. The mRNA levels of these genes in the positive control group showed no significant difference from those in the 0.79 g/kg Met-Met group (*p* > 0.05).

The relative gene expression levels of *caspase-2*, *-3*, *-7*, *-8*, *FasL*, *Apaf-1*, and *Bax* in the DI were downregulated with Met-Met supplementation to 1.44 or 1.84 g/kg (*p* < 0.05), respectively (Figure 3C). Meanwhile, the *caspase-9* mRNA level in the DI was significantly downregulated with an increase in the Met-Met level to 0.79 g/kg (*p* < 0.05), then had a plateau (*p* > 0.05). Moreover, the relative gene expression levels of *Bcl-2*, *IAP*, and *Mcl-1* in the DI were upregulated significantly by increased Met-Met levels to 1.44 g/kg diet (*p* < 0.05). Except for *caspase-3*, the relative expression levels of these genes in the positive control group showed no significant difference from those in the 0.79 g/kg Met-Met group (*p* > 0.05).

### 3.3. Effects of Supplemented Met-Met on the Relative Expression Levels of TJ Genes in Grass Carp Intestine

**Figure 4:**
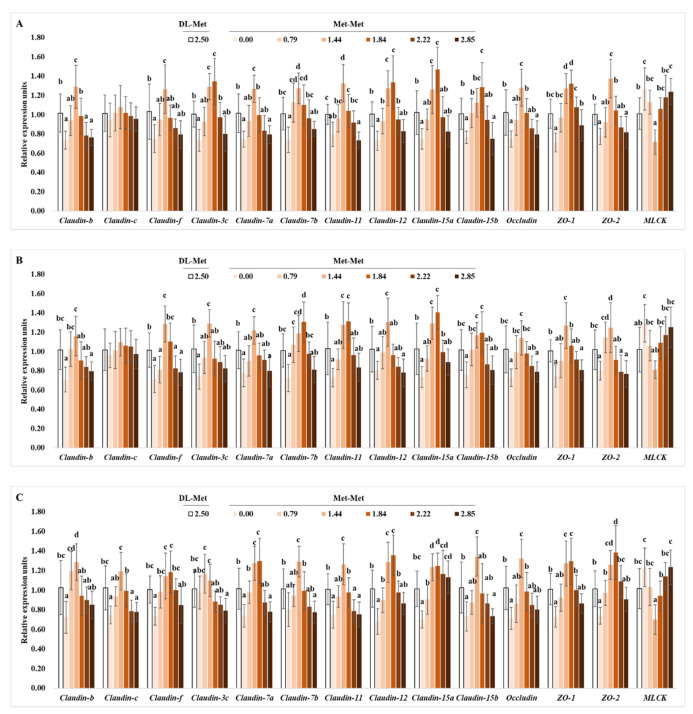
Effects of dietary DL-Met and Met-Met supplementation (g/kg diet) on relative expressions of tight-junction-related genes in the proximal intestine (**A**), mid intestine (**B**), and distal intestine (**C**) of juvenile grass carp. Data represent means of six fish in each group, error bars indicate S.D. ^a, b, c, d^ within a column, means without a common lowercase superscript differ (*p* < 0.05).

The present results showed that the mRNA levels of *claudin-b*, *-f*, *-7a*, *-7b*, *-11*, *-12*, *-15b*, *occludin*, *zonula occludens 1* (*ZO-1*), and *ZO-2* in the PI, MI, and DI, as well as those of *claudin-3c* and *claudin-15a* in the PI and MI, were upregulated significantly with Met-Met supplementation to 1.44 or 1.84 g/kg diet (*p* < 0.05), respectively, then declined with a further increase in Met-Met supplementation (*p* < 0.05) (Figure 4). Meanwhile, the relative gene expression level of *claudin-15a* in the DI was significantly upregulated with Met-Met supplementation to 1.44 g/kg diet (*p* < 0.05) and plateaued thereafter (*p* > 0.05). However, compared with the negative control group, Met-Met supplementation had no significant impacts on the relative expression levels of *claudin-c* in the PI, MI, and DI, or of *claudin-3c* in the DI (*p* < 0.05). Except for mid intestinal *claudin-f*, the relative expression levels of these genes in the positive control group (DL-Met-supplemented group) showed no significant difference from those in the group with 0.79 g/kg Met-Met (*p* > 0.05) (Figure 4).

## 4. Discussion

Our previous study found that Met-Met supplementation improved fish growth and intestinal immune function [12]. Besides the intestinal immune barrier, the intestinal physical barrier is another important barrier that could affect intestinal health in fish. Accordingly, the present study focuses on the effects of Met-Met supplementation on intestinal physical barrier functions in juvenile grass carp, as indicated by intestinal antioxidant capacity, cellular apoptosis, and intracellular tight junctions.

### 4.1. Optimal Met-Met Supplementation Enhanced Antioxidant Capacity Partly Related to Nrf2 Signaling in Fish Gut

As far as we know, the oxidative damage to lipids and proteins that is caused by an imbalance in the production and detoxification of ROS could impair cellular integrity and functionality [24]. Similar to all aerobic organisms, fish have also developed antioxidant defenses that include antioxidant enzymes, such as SOD, GPx, GST, GR, and CAT, as well as non-enzyme antioxidants such as GSH, to scavenge and detoxify ROS, thereby decreasing oxidative damage to cells [25]. In this study, we found, for the first time, that optimal Met-Met supplementation enhanced the capacities of T-AOC, AHR, and ASA; the activity of antioxidant enzymes including MnSOD, GPx, GST, GR, and CAT; and GSH content along with decreasing contents of ROS, MDA, and PC in juvenile grass carp intestines, suggesting that optimum Met-Met could increase antioxidant capacity in the fish gut, thereby decreasing oxidant damage. Similarly, optimal levels of Met-Met enhanced T-AOC and decreased MDA content in Nile tilapia liver [13] and in white leg shrimp plasma [15], as well as increasing hemolymph T-AOC and decreasing hepatopancreas MDA in Pacific white shrimp (*Litopenaeus vannamei*) [26]. Interestingly, the present results found that optimum Met-Met supplementation boosted the activity of MnSOD but not CuZnSOD in the intestine. This might be partly correlated to the expression of the *MnSOD* and *CuZnSOD* genes. In rats, the antioxidant enzyme activity was related to expression of the corresponding genes [27]. In this study, our data showed that Met-Met supplementation upregulated the relative expression of *MnSOD* but not the *CuZnSOD* gene in juvenile grass carp intestine, which could partly explain the change in MnSOD and CuZnSOD activity. Meanwhile, in this study, optimum Met-Met enhanced the relative gene expression levels of *CAT*, *GPx1a*, *GPx1b*, *GPx4a*, *GPx4b*, *GSTO1*, *GSTO2*, *GSTP2*, *GSTR*, and *GR* in three intestinal segments of juvenile grass carp, which might partly lead to the enhancement of their corresponding enzymes’ activity. However, in zebrafish, supplementation with 2.8 g/kg Met-Met increased gut *SOD2*, *CAT*, and *GPx1* gene expressions after treatment for 48 h, but decreased them after treatment for 20 d [28]. The supplemented dose of Met-Met might have led to different results in juvenile grass carp and zebrafish. In the present study, 2.85 g/kg Met-Met supplementation did not change the gene expressions of antioxidant enzymes compared with the negative control group but decreased the gene expressions when compared with the optimal Met-Met group. 

Another interesting result in this study was that optimum dietary Met-Met had different effects on various SOD and GST isoforms, as evident by the upregulated *MnSOD* and *GSTP2* mRNA levels but lack of change in the *CuZnSOD* and *GSTP1* mRNA levels in juvenile grass carp intestine. However, there was no more information about the effects of Met-Met supplementation on the various isoforms of antioxidant enzyme genes. The possible reasons for the difference might be as follows. Firstly, the various impacts of Met-Met supplementation on *MnSOD* and *CuZnSOD* gene expression might be partly correlated to the methionine accumulation in the mitochondria. To our knowledge, CuZnSOD is a major cytoplasmic antioxidant enzyme, whereas MnSOD is an important mitochondrial antioxidant enzyme in aerobic cells [29]. A study found that methionine is an evolutionarily selected antioxidant building block of respiratory chain complexes in living cells’ mitochondria [30]. Therefore, the accumulation of methionine in the mitochondria might partly contribute to the enhanced *MnSOD* gene expression in the optimal Met-Met group; however, this hypothesis needs further confirmation. Secondly, the various effects of Met-Met on the *GSTP1* and *GSTP2* gene expressions might be partly associated with Keap1. In rats, the knockout of *Keap1* upregulated the gene expression of *GSTP2* but not *GSTP1* in the liver [31]. In this study, optimum Met-Met downregulated *Keap1a* and *Keap1b* mRNA levels in juvenile grass carp intestines. Accordingly, the change in *GSTP1* and *GSTP2* might be partly due to the change of Keap1.

Furthermore, the most biologically relevant substrate of Keap1 is Nrf2, which serves as a major regulator of antioxidant enzymes or proteins [31]. In the present study, optimum Met-Met upregulated *Nrf2* relative gene expression. However, little information is currently available about the relationship between Met-Met and Nrf2 signaling. In human hepatic carcinoma cells, TOR inhibition decreased Nrf2 expression [32]. Our previous study found that optimum Met-Met enhanced total TOR and phosphorylated TOR levels in juvenile grass carp intestine [12], which might partly explain the improved expression of Nrf2 in this study.

### 4.2. Optimum Met-Met Supplementation Inhibited Intestinal Apoptosis in Fish

Additionally, apoptosis has also been implicated in contributing to compromised intestinal barrier function [33]. The death receptor pathway (including FasL/caspase-8) and the mitochondrial pathway (including Apaf-1/caspase-9) are the two main apoptotic pathways, which could be regulated by anti-apoptotic proteins such as Bcl-2, Mcl-1, and IAP and pro-apoptotic proteins such as Bax, and finally executed mainly by caspase-3 and -7 in mammals [34]. In this study, for the first time, we investigated the impacts of Met-Met on apoptosis in fish guts and found that optimum Met-Met down-regulated the relative gene expression levels of *caspase-2*, *-3*, *-7*, *-8*, and *-9*, as well as the pro-apoptotic-related proteins *FasL*, *Apaf-1*, and *Bax*, but increased the relative gene expression levels of the anti-apoptotic proteins *Bcl-2*, *Mcl-1*, and *IAP* in juvenile grass carp intestine, suggesting that Met-Met might suppress apoptosis related to the death receptor pathway and the mitochondrial pathway in animal gut. However, the involvement of Met-Met in decreasing cell apoptosis in fish has been ill-defined thus far. The Met-Met-decreased oxidative damage might partly contribute to the decrease in intestinal apoptosis. It has been known that oxidant damage could lead to cellular apoptosis [35]. As stated above, optimum Met-Met declined the ROS content and oxidative damage to lipids and proteins in fish intestine.

### 4.3. Optimum Met-Met Strengthened the Tight Junction in Fish Intestines

Intestinal physical barrier function is also correlated to intercellular tight junctions (TJ) in vertebrates, which are constituted by transmembrane proteins (e.g., claudins and occludin), as well as linker proteins (e.g., zonula occludens-1, -2) [33]. Decreased expression levels of occludin and ZO-1 would disturb the intestinal barrier function in fish [36]. Furthermore, the intestinal epithelial tight junction could be improved by inhibiting the activation of MLCK in mammals [37]. This study, for the first time, investigates the impacts of Met-Met on TJ proteins and MLCK in fish intestines. The present results found that optimum Met-Met upregulated *occludin, claudin-b, -c* (only in the DI), *-f*, *-3c* (not in the DI), *-7a*, *-7b*, *-11*, *-12*, *-15a*, *-15b*, *ZO-1*, and *ZO-2* mRNA levels and down-regulated MLCK mRNA levels in juvenile grass carp intestines, suggesting that optimum Met-Met supplementation could improve the tight junctions in fish intestines. To our knowledge, there is no more information about the regulation of intracellular tight junction proteins by Met-Met in fish intestines. In domestic pigeons (*Columba livia*), a similar result showed that Met-Met supplementation also increased the protein expression of claudin-1 and ZO-1 in the jejunum [20], demonstrating that optimal Met-Met was beneficial for the intestinal tight junctions of animals.

Interestingly, we found that Met-Met supplementation did not affect the relative gene expression of claudin-c in the PI and MI of grass carp, but increased it in the DI. The mechanisms underlying these impacts are not well characterized, but might be partly correlated with IL-8. A study in humans showed that IL-8 could stimulate the release of cortisol [38]. In goldfish, cortisol decreased the claudin-c mRNA level in gill epithelia [35]. Our previous study observed that optimal Met-Met downregulated the relative mRNA level of IL-8 only in the DI and not in the PI and MI. Therefore, the increased claudin-c in the DI might be attributed to the downregulated distal intestine IL-8 by optimal Met-Met. However, the detailed mechanisms await further characterization.

### 4.4. The Comparison between the Influences of Met-Met and DL-Met on the Intestinal Physical Barrier in Fish

As elucidated above, Met-Met supplementation at 1.44–1.84 g/kg could enhance the intestinal physical barrier function, probably by enhancing antioxidant capacity, decreasing excessive cellular apoptosis, and improving intracellular tight junctions, in juvenile grass carp. When compared with the positive control (DL-Met 2.50 g/kg), the beneficial effects of 1.44–1.84 g/kg Met-Met on intestinal antioxidant capacity, cellular apoptosis, and intracellular tight junctions were equal or better. The possible reasons might be as follows. Firstly, it was found that the absorption rates of Met-Met were greater than those of crystalline methionine in the intestine of rats [39]. Secondly, the asynchronous absorption of dietary crystalline methionine and protein-bound methionine could reduce the protein synthesis efficiency in fish [10]. Moreover, in comparison with crystalline methionine, methionine-containing dipeptides showed greater protein synthesis efficiency in bovine mammary epithelial cells [40]. Therefore, the stronger influence of Met-Met on fish intestinal barrier function might be related to the fact that dietary Met-Met had better absorption rates and protein synthesis efficiency than DL-Met. This finding indicated that Met-Met might be a good source of methionine for fish.

### 4.5. The Optimum Levels of Met-Met for Juvenile Grass Carp

With the continuous advances in the understanding of cell and organism metabolism, determining the nutrients requirement or optimal supplementation of feed additives based on various aspects in addition to fish growth is useful for aquaculture. Thus, in this study, we determined the optimum levels of Met-Met supplementation on the basis of intestinal antioxidant parameters, and found that the optimum levels of Met-Met for juvenile grass carp ranged from 1.52 to 1.73 g/kg diet (with methionine at 4.26 g/kg and cysteine at 3.77 g/kg in the diet) based on the MDA content and T-AOC in the three intestinal segments (Table 7), which were close to those based on growth performance and intestinal immunity (1.61–1.68 g/kg diet) [12]. Similar results were found in leucine requirements for young grass carp, which showed that the leucine requirements for young grass carp based on intestinal antioxidant capacity were close to those based on growth performance and intestinal immunity [41,42]. These results indicated that the improved grass carp growth by Met-Met supplementation, like Leu, partly resulted from maintaining the fish intestinal health. It is reported that the health of fish affects consumers’ willingness to pay more for fish [43]. Meanwhile, fish health is a major concern of fish farmers, and could affect fish flesh quality [44]. Accordingly, for aquaculture systems, it is better to use a dose of Met-Met at 1.68–1.73 g/kg diet, which was better for grass carp intestinal health. 

## 5. Conclusions

**Figure 5:**
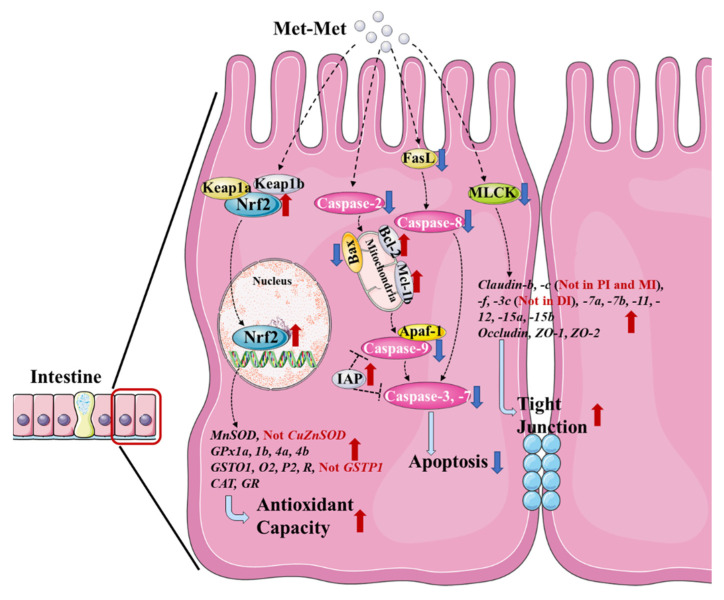
General summary for the effects of Met-Met on the intestinal physical barrier in fish.

As summarized in Figure 5, this study found that optimum levels of Met-Met increased the antioxidant capacity of fish intestine, as evident by the enhanced activities and gene expressions of MnSOD, CAT, GPx, GST, and GR, which were probably correlated with the change in Nrf2/Keap1 signaling; decreased intestinal apoptosis, as evident by downregulated pro-apoptotic-related proteins and upregulated anti-apoptotic proteins; and improved intestinal tight junctions, as evident by the enhanced gene expressions of tight junction proteins (claudins, occludin, and ZOs), thereby being helpful for enhancing fish intestinal physical barrier function. However, supplementation with Met-Met showed different effects on various *SOD* and *GST* isoforms in the intestine, and on *claudin-c* and *-3c* in various intestinal segments with unknown mechanisms. Additionally, the positive influences of optimal Met-Met on improving intestinal physical barrier function were superior to those of DL-Met in fish. The optimum levels of Met-Met based on antioxidant parameters (MDA contents and T-AOC in the three intestinal segments) were estimated to be 1.52–1.73 g/kg diet (with methionine at 4.26 g/kg and cysteine at 3.77 g/kg in the diet) for juvenile grass carp.

## Figures and Tables

**Table 1:** Formulation and nutrients content of the basal diet.

Ingredients	Content (%)	Nutrients	Content (%)
Fish meal	3.00	Crude protein ^d^	32.64
Soybean meal	36.90	Crude lipid ^d^	4.45
Cottonseed meal	22.72	Methionine ^d^	0.43
Fish oil	2.75	Cysteine ^d^	0.38
Soybean oil	1.25	n-3 ^e^	1.05
Wheat flour	22.33	n-6 ^e^	0.97
Vitamin premix ^a^	1.00	Available phosphorus ^f^	0.84
Mineral premix ^b^	1.00		
Ca(H_2_PO_4_)_2_	2.98		
L-Lys (98.5%)	0.35		
L-Thr (98.5%)	0.17		
Carboxymethyl cellulose	2.00		
Choline chloride (50%)	0.50		
Ethoxyquin (30%)	0.05		
Met-Met/DL-Met premix ^c^	3.00		

^a^ Per kilogram of vitamin premix (g/kg): retinyl acetate (500,000 IU/g), 2.10; cholecalciferol (500,000 IU/g), 0.40; D, L-α-tocopherol acetate (50%), 12.58; menadione (22.9%), 0.83; cyanocobalamin (1%), 0.94; D-biotin (2%), 0.75; folic acid (95%), 0.42; thiamine nitrate (98%), 0.09; ascorhyl acetate (95%), 4.31; niacin (99%), 4.04; meso-inositol (98%), 19.39; calcium-D-pantothenate (98%), 3.85; riboflavin (80%), 0.73; pyridoxine hydrochloride (98%), 0.62. All ingredients were diluted with corn starch to 1 kg. ^b^ Per kilogram of mineral premix (g/kg): MnSO_4_.H_2_O (31.8% Mn), 2.6590; MgSO_4_.H_2_O (15.0% Mg), 200.0000; FeSO_4_.H_2_O (30.0% Fe), 12.2500; ZnSO_4_.H_2_O (34.5% Zn), 8.2460; CuSO_4_.5H_2_O (25.0% Cu), 0.9560; KI (76.9% I), 0.0650; Na_2_SeO_3_ (44.7% Se), 0.0168. All ingredients were diluted with corn starch to 1 kg. ^c^ Met-Met/DL-Met premix was added to obtain DL-Met or graded levels of Met-Met, and the amount of glycine and corn starch was reduced to compensate. The per-kilogram of premix composition from diet 1 was 83.33 g DL-Met, 317.67 g glycine and 599.00 g corn starch. Premix composition per kilogram from diet 2 to 7 was as follows: Met-Met 0.00, 33.33, 50.00, 66.67, 83.33, 100.00 g, glycine 360.00, 342.00, 333.00, 324.00, 315.00, 306.00 g and corn starch 640.00, 624.67, 617.00, 609.33, 601.67, 594.00 g, respectively. ^d^ Crude protein, crude lipid, methionine, and cysteine contents were the measured values. The methionine concentrations from diet 1 to 7 were analyzed to be 6.71, 4.26, 5.03, 5.75, 6.23, 6.70, and 7.42 g/kg by the method of high-performance liquid-chromatographic (HPLC) as described by our previous study, Su et al. [12]. ^e^ n-3 and n-6 contents were referenced to Zeng et al. [22] and calculated according to NRC [23]. ^f^ Available phosphorus was calculated according to NRC [23].

**Table 2:** Real-time PCR primer sequences.

Target Gene	Primer Sequence FORWARD (5′→3′)	Primer Sequence Reverse (5′→3′)	Temperature (°C)	Accession Number
*occludin*	TATCTGTATCACTACTGCGTCG	CATTCACCCAATCCTCCA	59.4	KF193855
*ZO-1*	CGGTGTCTTCGTAGTCGG	CAGTTGGTTTGGGTTTCAG	59.4	KJ000055
*ZO-2*	TACAGCGGGACTCTAAAATGG	TCACACGGTCGTTCTCAAAG	60.3	KM112095
*claudin-b*	GAGGGAATCTGGATGAGC	ATGGCAATGATGGTGAGA	57.0	KF193860
*claudin-c*	GAGGGAATCTGGATGAGC	CTGTTATGAAAGCGGCAC	59.4	KF193859
*claudin-f*	GCTGGAGTTGCCTGTCTTATTC	ACCAATCTCCCTCTTTTGTGTC	57.1	KM112097
*claudin-3c*	ATCACTCGGGACTTCTA	CAGCAAACCCAATGTAG	57.0	KF193858
*claudin-7a*	ACTTACCAGGGACTGTGGATGT	CACTATCATCAAAGCACGGGT	59.3	KT625604
*claudin-7b*	CTAACTGTGGTGGTGATGAC	AACAATGCTACAAAGGGCTG	59.3	KT445866
*claudin-11*	TCTCAACTGCTCTGTATCACTGC	TTTCTGGTTCACTTCCGAGG	62.3	KT445867
*claudin-12*	CCCTGAAGTGCCCACAA	GCGTATGTCACGGGAGAA	55.4	KF998571
*claudin-15a*	TGCTTTATTTCTTGGCTTTC	CTCGTACAGGGTTGAGGTG	59.0	KF193857
*claudin-15b*	AGTGTTCTAAGATAGGAGGGGAG	AGCCCTTCTCCGATTTCAT	62.3	KT757304
*FasL*	AGGAAATGCCCGCACAAATG	AACCGCTTTCATTGACCTGGAG	61.4	KT445873
*Bcl-2*	AGGAAAATGGAGGTTGGGAT	CTGAGCAAAAAAGGCGATG	60.3	JQ713862.1
*Mcl-1b*	TGGAAAGTCTCGTGGTAAAGCA	ATCGCTGAAGATTTCTGTTGCC	58.4	KT757307
*Bax*	CATCTATGAGCGGGTTCGTC	TTTATGGCTGGGGTCACACA	60.3	JQ793788.1
*Apaf-1*	AAGTTCTGGAGCCTGGACAC	AACTCAAGACCCCACAGCAC	61.4	KM279717
*IAP*	CACAATCCTGGTATGCGTCG	GGGTAATGCCTCTGGTGCTC	58.4	FJ593503.1
*caspase-2*	CGCTGTTGTGTGTTTACTGTCTCA	ACGCCATTATCCATCTCCTCTC	60.3	KT757313
*caspase-3*	GCTGTGCTTCATTTGTTTG	TCTGAGATGTTATGGCTGTC	55.9	JQ793789
*caspase-7*	GCCATTACAGGATTGTTTCACC	CCTTATCTGTGCCATTGCGT	57.1	KT625601
*caspase-8*	ATCTGGTTGAAATCCGTGAA	TCCATCTGATGCCCATACAC	59.0	KM016991
*caspase-9*	CTGTGGCGGAGGTGAGAA	GTGCTGGAGGACATGGGAAT	59.0	JQ793787
*CuZnSOD*	CGCACTTCAACCCTTACA	ACTTTCCTCATTGCCTCC	61.5	GU901214
*MnSOD*	ACGACCCAAGTCTCCCTA	ACCCTGTGGTTCTCCTCC	60.4	GU218534
*CAT*	GAAGTTCTACACCGATGAGG	CCAGAAATCCCAAACCAT	58.7	FJ560431
*GPx1a*	GGGCTGGTTATTCTGGGC	AGGCGATGTCATTCCTGTTC	61.5	EU828796
*GPx1b*	TTTTGTCCTTGAAGTATGTCCGTC	GGGTCGTTCATAAAGGGCATT	60.3	KT757315
*GPx4a*	TACGCTGAGAGAGGTTTACACAT	CTTTTCCATTGGGTTGTTCC	60.4	KU255598
*GPx4b*	CTGGAGAAATACAGGGGTTACG	CTCCTGCTTTCCGAACTGGT	60.3	KU255599
*GSTR*	TCTCAAGGAACCCGTCTG	CCAAGTATCCGTCCCACA	58.4	EU107283
*GSTP1*	ACAGTTGCCCAAGTTCCAG	CCTCACAGTCGTTTTTTCCA	59.3	KM112099
*GSTP2*	TGCCTTGAAGATTATGCTGG	GCTGGCTTTTATTTCACCCT	59.3	KP125490
*GSTO1*	GGTGCTCAATGCCAAGGGAA	CTCAAACGGGTCGGATGGAA	58.4	KT757314
*GSTO2*	CTGCTCCCATCAGACCCATTT	TCTCCCCTTTTCTTGCCCATA	61.4	KU245630
*GR*	GTGTCCAACTTCTCCTGTG	ACTCTGGGGTCCAAAACG	59.4	JX854448
*Nrf2*	CTGGACGAGGAGACTGGA	ATCTGTGGTAGGTGGAAC	62.5	KF733814
*Keap1a*	TTCCACGCCCTCCTCAA	TGTACCCTCCCGCTATG	63.0	KF811013
*Keap1b*	TCTGCTGTATGCGGTGGGC	CTCCTCCATTCATCTTTCTCG	57.9	KJ729125
*β-actin*	GGCTGTGCTGTCCCTGTA	GGGCATAACCCTCGTAGAT	61.4	M25013

ZO, zonula occludens; FasL, fatty acid synthetase ligand; Bcl-2, B-cell lymphoma protein-2; Mcl-1b, myeloid cell leukemia-1b; Bax, Bcl-2-associated X protein; Apaf-1, apoptotic protease-activating factor-1; IAP, inhibitor of apoptosis proteins; caspase, cysteinyl aspartic acid-protease; CuZnSOD, copper/zinc superoxide dismutase; MnSOD, manganese superoxide dismutase; CAT, catalase; GPx, glutathione peroxidase; GST, glutathione-S-transferase; GR, glutathione reductase; Nrf2, NF-E2-related factor 2; Keap1, Kelch-like-ECH-associated protein 1.

**Table 3:** Effects of dietary DL-Met and Met-Met supplementation (g/kg diet) on antioxidant-related parameters in three intestinal segments of juvenile grass carp.

	DL-Met	Met-Met					
	2.50	0.00	0.79	1.44	1.84	2.22	2.85
Proximal intestine							
T-SOD	16.65 ± 1.05 ^bc^	14.74 ± 0.82 ^a^	16.07 ± 0.62 ^b^	17.55 ± 0.88 ^cd^	18.64 ± 1.06 ^d^	17.29 ± 1.15 ^c^	16.51 ± 1.01 ^bc^
CuZnSOD	10.29 ± 0.89	10.05 ± 0.85	10.15 ± 0.74	10.83 ± 0.77	10.80 ± 0.85	10.45 ± 0.75	10.13 ± 0.84
MnSOD	6.36 ± 0.38 ^bc^	4.69 ± 0.39 ^a^	5.93 ± 0.35 ^b^	6.72 ± 0.45 ^c^	7.84 ± 0.66 ^d^	6.84 ± 0.56 ^c^	6.39 ± 0.56 ^bc^
CAT	2.46 ± 0.19 ^bc^	1.97 ± 0.18 ^a^	2.36 ± 0.14 ^b^	2.63 ± 0.20 ^c^	2.62 ± 0.13 ^c^	2.64 ± 0.26 ^c^	2.61 ± 0.16 ^c^
GPx	150.94 ± 11.12 ^c^	103.17 ± 7.81 ^a^	129.66 ± 8.18 ^b^	146.66 ± 8.46 ^c^	156.66 ± 9.80 ^c^	157.77 ± 8.17 ^c^	156.91 ± 9.00 ^c^
GST	59.26 ± 3.43 ^c^	42.35 ± 2.74 ^a^	58.59 ± 4.58 ^c^	64.34 ± 4.92 ^d^	57.61 ± 2.60 ^c^	54.68 ± 4.23 ^bc^	50.95 ± 3.19 ^b^
GR	51.07 ± 2.92 ^cd^	36.66 ± 2.32 ^a^	48.10 ± 2.91 ^bc^	58.05 ± 3.25 ^e^	62.93 ± 4.40 ^f^	52.53 ± 4.10 ^d^	46.15 ± 3.52 ^b^
GSH	7.05 ± 0.36 ^c^	4.06 ± 0.37 ^a^	6.38 ± 0.22 ^b^	7.90 ± 0.42 ^d^	6.98 ± 0.47 ^c^	6.24 ± 0.46 ^b^	6.01 ± 0.42 ^b^
Mid intestine							
T-SOD	16.11 ± 0.65 ^c^	12.69 ± 0.79 ^a^	15.85 ± 0.64 ^c^	16.50 ± 0.90 ^c^	18.34 ± 0.81 ^d^	16.32 ± 0.77 ^c^	14.76 ± 0.75 ^b^
CuZnSOD	9.25 ± 0.54	8.99 ± 0.82	9.26 ± 0.67	9.57 ± 0.42	9.69 ± 0.49	9.64 ± 0.39	8.98 ± 0.69
MnSOD	6.86 ± 0.56 ^c^	3.70 ± 0.30 ^a^	6.60 ± 0.25 ^c^	6.93 ± 0.55 ^c^	8.65 ± 0.70 ^d^	6.67 ± 0.53 ^c^	5.79 ± 0.41 ^b^
CAT	3.60 ± 0.17 ^b^	3.00 ± 0.18 ^a^	3.66 ± 0.13 ^b^	3.57 ± 0.10 ^b^	3.63 ± 0.27 ^b^	3.63 ± 0.13 ^b^	3.60 ± 0.14 ^b^
GPx	146.70 ± 9.91 ^b^	108.59 ± 7.15 ^a^	137.27 ± 7.03 ^b^	158.61 ± 10.37 ^c^	179.94 ± 9.84 ^d^	147.05 ± 10.62 ^b^	136.25 ± 10.62 ^b^
GST	60.54 ± 2.18 ^e^	46.15 ± 3.24 ^a^	55.81 ± 1.47 ^c^	61.58 ± 3.12 ^e^	59.00 ± 2.50 ^de^	56.83 ± 1.16 ^cd^	52.92 ± 1.67 ^b^
GR	61.42 ± 3.39 ^d^	37.91 ± 2.15 ^a^	51.39 ± 4.35 ^bc^	67.91 ± 5.09 ^e^	60.48 ± 5.09 ^d^	55.83 ± 3.71 ^c^	49.70 ± 2.87 ^b^
GSH	7.73 ± 0.71 ^c^	4.81 ± 0.41 ^a^	5.91 ± 0.31 ^b^	7.40 ± 0.65 ^c^	7.93 ± 0.74 ^c^	6.24 ± 0.62 ^b^	5.84 ± 0.43 ^b^
Distal intestine							
T-SOD	16.57 ± 0.79 ^bc^	14.35 ± 0.42 ^b^	16.31 ± 0.69 ^a^	17.54 ± 1.11 ^cd^	18.13 ± 0.37 ^d^	16.97 ± 0.84 ^bc^	16.13 ± 1.12 ^b^
CuZnSOD	9.54 ± 0.67	9.46 ± 0.41	9.55 ± 0.29	9.64 ± 0.51	9.60 ± 0.74	9.52 ± 0.47	9.47 ± 0.92
MnSOD	7.03 ± 0.32 ^bc^	4.89 ± 0.41 ^a^	6.76 ± 0.52 ^b^	7.90 ± 0.70 ^d^	8.53 ± 0.50 ^e^	7.45 ± 0.65 ^cd^	6.66 ± 0.48 ^b^
CAT	2.57 ± 0.22 ^bc^	2.30 ± 0.13 ^a^	2.55 ± 0.09 ^bc^	2.70 ± 0.14 ^c^	2.48 ± 0.19 ^ab^	2.44 ± 0.19 ^ab^	2.37 ± 0.14 ^ab^
GPx	126.59 ± 8.04 ^d^	98.11 ± 4.86 ^a^	117.42 ± 5.69 ^c^	137.53 ± 8.66 ^e^	128.77 ± 5.39 ^d^	117.85 ± 8.47 ^c^	107.95 ± 7.18 ^b^
GST	61.64 ± 4.96 ^c^	49.32 ± 1.59 ^a^	57.01 ± 1.11 ^b^	63.04 ± 1.56 ^c^	65.45 ± 3.67 ^c^	56.51 ± 3.79 ^b^	52.37 ± 3.43 ^a^
GR	51.98 ± 4.34 ^c^	38.98 ± 3.11 ^a^	47.14 ± 3.65 ^b^	57.81 ± 3.44 ^d^	64.57 ± 2.99 ^e^	52.42 ± 2.59 ^c^	46.32 ± 2.19 ^b^
GSH	7.15 ± 0.37 ^c^	3.81 ± 0.18 ^a^	4.92 ± 0.24 ^b^	7.44 ± 0.73 ^c^	8.33 ± 0.53 ^d^	6.99 ± 0.60 ^c^	5.41 ± 0.36 ^b^

Values are means and standard deviations (*n* = 6). ^a, b, c, d, e, f^ within a row, means without a common lowercase superscript differ (*p* < 0.05). T-SOD, total superoxide dismutase (U/mg protein); CuZnSOD, copper/zinc superoxide dismutase (U/mg protein); MnSOD, manganese superoxide dismutase (U/mg protein); CAT, catalase (U/mg protein); GPx, glutathione peroxidase (U/mg protein); GST, glutathione-S-transferase (U/mg protein); GR, glutathione reductase (U/g protein); GSH, glutathione (mg/g protein).

**Table 4:** Effects of dietary DL-Met and Met-Met supplementation (g/kg diet) on relative gene expressions of antioxidant enzymes and Nrf2 signaling in the proximal intestine of juvenile grass carp.

	DL-Met	Met-Met					
	2.50	0.00	0.79	1.44	1.84	2.22	2.85
*CuZnSOD*	1.02 ± 0.20	0.99 ± 0.18	1.07 ± 0.15	1.08 ± 0.14	1.08 ± 0.16	1.05 ± 0.16	1.04 ± 0.11
*MnSOD*	1.02 ± 0.22 ^b^	0.71 ± 0.16 ^a^	0.95 ± 0.11 ^b^	1.27 ± 0.13 ^c^	1.29 ± 0.17 ^c^	1.00 ± 0.21 ^b^	0.84 ± 0.14 ^ab^
*CAT*	1.01 ± 0.11 ^bc^	0.71 ± 0.13 ^a^	0.88 ± 0.10 ^ab^	1.15 ± 0.25 ^c^	1.11 ± 0.22 ^c^	1.12 ± 0.13 ^c^	1.07 ± 0.19 ^bc^
*GPX1a*	1.01 ± 0.17 ^bc^	0.69 ± 0.13 ^a^	0.84 ± 0.16 ^ab^	1.04 ± 0.20 ^bc^	1.12 ± 0.17 ^c^	1.06 ± 0.16 ^c^	0.95 ± 0.13 ^bc^
*GPx1b*	1.01 ± 0.17 ^b^	0.71 ± 0.14 ^a^	1.00 ± 0.22 ^b^	1.12 ± 0.13 ^b^	1.13 ± 0.15 ^b^	1.09 ± 0.14 ^b^	0.95 ± 0.12 ^b^
*GPx4a*	1.02 ± 0.19 ^b^	0.75 ± 0.12 ^a^	0.96 ± 0.16 ^ab^	1.37 ± 0.27 ^c^	1.02 ± 0.19 ^b^	0.85 ± 0.13 ^ab^	0.74 ± 0.13 ^a^
*GPx4b*	1.01 ± 0.18 ^b^	0.73 ± 0.13 ^a^	0.93 ± 0.16 ^ab^	1.27 ± 0.29 ^c^	0.89 ± 0.16 ^ab^	0.81 ± 0.09 ^ab^	0.79 ± 0.13 ^a^
*GSTo1*	1.02 ± 0.23 ^bc^	0.74 ± 0.14 ^a^	1.00 ± 0.12 ^bc^	1.29 ± 0.15 ^d^	1.34 ± 0.25 ^d^	1.16 ± 0.20 ^cd^	0.88 ± 0.17 ^ab^
*GSTo2*	1.01 ± 0.17 ^b^	0.74 ± 0.14 ^a^	1.00 ± 0.23 ^b^	1.23 ± 0.17 ^c^	1.36 ± 0.25 ^c^	0.94 ± 0.18 ^ab^	0.83 ± 0.15 ^ab^
*GSTp1*	1.01 ± 0.13	0.96 ± 0.13	1.04 ± 0.13	1.08 ± 0.20	1.02 ± 0.10	1.12 ± 0.15	1.09 ± 0.22
*GSTp2*	1.02 ± 0.25 ^b^	0.73 ± 0.15 ^a^	0.93 ± 0.13 ^ab^	1.41 ± 0.12 ^c^	1.05 ± 0.22 ^b^	0.84 ± 0.16 ^ab^	0.79 ± 0.15 ^a^
*GSTR*	1.02 ± 0.20 ^b^	0.75 ± 0.14 ^a^	0.99 ± 0.23 ^ab^	1.35 ± 0.22 ^c^	1.06 ± 0.22 ^b^	0.98 ± 0.11 ^ab^	0.86 ± 0.16 ^ab^
*GR*	1.01 ± 0.17 ^bc^	0.72 ± 0.19 ^a^	0.89 ± 0.21 ^ab^	1.16 ± 0.29 ^c^	1.20 ± 0.21 ^c^	0.88 ± 0.14 ^ab^	0.79 ± 0.17 ^ab^
*Nrf2*	1.02 ± 0.22 ^cd^	0.59 ± 0.10 ^a^	1.06 ± 0.17 ^cd^	1.16 ± 0.22 ^d^	1.04 ± 0.19 ^cd^	0.90 ± 0.17 ^bc^	0.79 ± 0.19 ^ab^
*Keap1a*	1.01 ± 0.17 ^ab^	1.58 ± 0.19 ^c^	1.02 ± 0.18 ^ab^	0.82 ± 0.19 ^a^	1.03 ± 0.20 ^ab^	1.12 ± 0.21 ^b^	1.22 ± 0.22 ^b^
*Keap1b*	1.01 ± 0.13 ^ab^	1.28 ± 0.15 ^c^	1.12 ± 0.17 ^bc^	0.85 ± 0.13 ^a^	1.03 ± 0.16 ^ab^	1.13 ± 0.13 ^bc^	1.20 ± 0.19 ^bc^

Values are means and standard deviations (*n* = 6). ^a, b, c, d^ within a row, means without a common lowercase superscript differ (*p* < 0.05). CuZnSOD, copper/zinc superoxide dismutase; MnSOD, manganese superoxide dismutase; CAT, catalase; GPx, glutathione peroxidase; GST, glutathione-S-transferase; GR, glutathione reductase; Nrf2, NF-E2-related factor 2; Keap1, Kelch-like-ECH-associated protein 1.

**Table 5:** Effects of dietary DL-Met and Met-Met supplementation (g/kg diet) on relative gene expressions of antioxidant enzymes and Nrf2 signaling in the mid intestine of juvenile grass carp.

	DL-Met	Met-Met					
	2.50	0.00	0.79	1.44	1.84	2.22	2.85
*CuZnSOD*	1.02 ± 0.21	0.99 ± 0.13	1.07 ± 0.18	1.09 ± 0.16	1.02 ± 0.17	1.04 ± 0.24	1.03 ± 0.19
*MnSOD*	1.01 ± 0.12 ^b^	0.80 ± 0.06 ^a^	0.97 ± 0.20 ^ab^	1.26 ± 0.19 ^c^	0.91 ± 0.13 ^ab^	0.89 ± 0.13 ^ab^	0.81 ± 0.14 ^a^
*CAT*	1.02 ± 0.21 ^b^	0.71 ± 0.10 ^a^	0.91 ± 0.13 ^b^	0.96 ± 0.12 ^b^	1.07 ± 0.12 ^b^	1.02 ± 0.13 ^b^	1.07 ± 0.17 ^b^
*GPX1a*	1.03 ± 0.22 ^b^	0.74 ± 0.14 ^a^	0.86 ± 0.09 ^ab^	1.26 ± 0.18 ^c^	1.37 ± 0.24 ^c^	1.03 ± 0.19 ^b^	0.84 ± 0.11 ^ab^
*GPx1b*	1.01 ± 0.18 ^bc^	0.74 ± 0.09 ^a^	1.06 ± 0.17 ^bc^	1.21 ± 0.19 ^cd^	1.29 ± 0.18 ^d^	0.92 ± 0.18 ^ab^	0.77 ± 0.13 ^a^
*GPx4a*	1.01 ± 0.16 ^bc^	0.71 ± 0.14 ^a^	1.04 ± 0.21 ^bc^	1.22 ± 0.17 ^c^	1.10 ± 0.21 ^c^	0.85 ± 0.12 ^ab^	0.79 ± 0.12 ^a^
*GPx4b*	1.01 ± 0.16 ^bc^	0.71 ± 0.14 ^a^	0.83 ± 0.11 ^ab^	1.13 ± 0.20 ^c^	1.37 ± 0.16 ^d^	0.94 ± 0.13 ^bc^	0.85 ± 0.18 ^ab^
*GSTo1*	1.01 ± 0.13 ^b^	0.73 ± 0.10 ^a^	1.02 ± 0.18 ^b^	1.27 ± 0.14 ^c^	1.30 ± 0.21 ^c^	1.03 ± 0.21 ^b^	0.84 ± 0.16 ^ab^
*GSTo2*	1.02 ± 0.22 ^ab^	0.79 ± 0.15 ^a^	1.02 ± 0.16 ^ab^	1.14 ± 0.23 ^bc^	1.26 ± 0.15 ^c^	1.00 ± 0.20 ^ab^	0.83 ± 0.13 ^a^
*GSTp1*	1.01 ± 0.20	0.99 ± 0.14	1.07 ± 0.20	1.09 ± 0.19	1.06 ± 0.20	1.02 ± 0.15	1.02 ± 0.13
*GSTp2*	1.01 ± 0.18 ^bc^	0.73 ± 0.14 ^a^	0.99 ± 0.11 ^bc^	1.13 ± 0.23 ^c^	0.98 ± 0.13 ^bc^	0.87 ± 0.17 ^ab^	0.83 ± 0.11 ^ab^
*GSTR*	1.02 ± 0.19 ^b^	0.73 ± 0.12 ^a^	0.92 ± 0.16 ^ab^	1.30 ± 0.20 ^c^	1.38 ± 0.27 ^c^	0.99 ± 0.16 ^b^	0.82 ± 0.07 ^ab^
*GR*	1.02 ± 0.21 ^bc^	0.66 ± 0.13 ^a^	1.17 ± 0.19 ^cd^	1.31 ± 0.21 ^d^	1.00 ± 0.16 ^bc^	0.90 ± 0.14 ^b^	0.82 ± 0.11 ^ab^
*Nrf2*	1.00 ± 0.10 ^bc^	0.74 ± 0.11 ^a^	1.14 ± 0.18 ^cd^	1.28 ± 0.18 ^d^	1.00 ± 0.10 ^bc^	0.92 ± 0.14 ^b^	0.86 ± 0.08 ^ab^
*Keap1a*	1.02 ± 0.26 ^bc^	1.36 ± 0.14 ^d^	0.85 ± 0.17 ^ab^	0.73 ± 0.12 ^a^	1.09 ± 0.12 ^c^	1.19 ± 0.16 ^cd^	1.21 ± 0.19 ^cd^
*Keap1b*	1.01 ± 0.19 ^ab^	1.27 ± 0.20 ^c^	0.97 ± 0.13 ^a^	0.88 ± 0.13 ^a^	1.20 ± 0.17 ^bc^	1.23 ± 0.22 ^c^	1.24 ± 0.16 ^c^

Values are means and standard deviations (*n* = 6). ^a, b, c, d^ within a row, means without a common lowercase superscript differ (*p* < 0.05). CuZnSOD, copper/zinc superoxide dismutase; MnSOD, manganese superoxide dismutase; CAT, catalase; GPx, glutathione peroxidase; GST, glutathione-S-transferase; GR, glutathione reductase; Nrf2, NF-E2-related factor 2; Keap1, Kelch-like-ECH-associated protein 1.

**Table 6:** Effects of dietary DL-Met and Met-Met supplementation (g/kg diet) on relative gene expressions of antioxidant enzymes and Nrf2 signaling in the distal intestine of juvenile grass carp.

	DL-Met	Met-Met					
	2.50	0.00	0.79	1.44	1.84	2.22	2.85
*CuZnSOD*	1.03 ± 0.24	1.00 ± 0.20	1.07 ± 0.13	1.14 ± 0.23	1.06 ± 0.24	1.02 ± 0.20	1.01 ± 0.19
*MnSOD*	1.01 ± 0.18 ^b^	0.74 ± 0.12 ^a^	0.93 ± 0.08 ^ab^	1.29 ± 0.19 ^c^	0.94 ± 0.19 ^ab^	0.86 ± 0.15 ^ab^	0.83 ± 0.14 ^ab^
*CAT*	1.01 ± 0.15 ^ab^	0.86 ± 0.21 ^a^	1.00 ± 0.13 ^ab^	1.17 ± 0.22 ^bc^	1.27 ± 0.20 ^c^	0.93 ± 0.15 ^a^	0.84 ± 0.08 ^a^
*GPX1a*	1.01 ± 0.15 ^bc^	0.71 ± 0.12 ^a^	1.00 ± 0.16 ^bc^	1.18 ± 0.09 ^cd^	1.27 ± 0.22 ^d^	1.09 ± 0.17 ^cd^	0.82 ± 0.15 ^ab^
*GPx1b*	1.02 ± 0.22 ^bc^	0.74 ± 0.15 ^a^	1.00 ± 0.13 ^abc^	1.26 ± 0.32 ^c^	1.27 ± 0.19 ^c^	1.08 ± 0.25 ^bc^	0.86 ± 0.14 ^ab^
*GPx4a*	1.00 ± 0.11 ^c^	0.68 ± 0.10 ^a^	0.83 ± 0.14 ^ab^	1.26 ± 0.14 ^d^	1.01 ± 0.14 ^c^	0.89 ± 0.15 ^bc^	0.74 ± 0.13 ^ab^
*GPx4b*	1.01 ± 0.15 ^b^	0.74 ± 0.14 ^a^	0.92 ± 0.15 ^ab^	1.39 ± 0.27 ^c^	1.07 ± 0.17 ^b^	0.87 ± 0.16 ^ab^	0.77 ± 0.11 ^a^
*GSTo1*	1.02 ± 0.23 ^b^	0.73 ± 0.13 ^a^	0.84 ± 0.13 ^ab^	1.28 ± 0.23 ^c^	0.99 ± 0.19 ^b^	0.81 ± 0.20 ^ab^	0.73 ± 0.12 ^a^
*GSTo2*	1.02 ± 0.23 ^bc^	0.78 ± 0.15 ^a^	1.03 ± 0.13 ^bc^	1.23 ± 0.12 ^cd^	1.25 ± 0.23 ^d^	0.97 ± 0.16 ^ab^	0.80 ± 0.15 ^a^
*GSTp1*	1.01 ± 0.18	1.02 ± 0.14	1.05 ± 0.16	1.10 ± 0.20	1.00 ± 0.14	1.06 ± 0.21	0.98 ± 0.15
*GSTp2*	1.01 ± 0.18 ^bc^	0.77 ± 0.18 ^a^	0.95 ± 0.15 ^ab^	1.20 ± 0.21 ^c^	0.98 ± 0.15 ^b^	0.83 ± 0.13 ^ab^	0.75 ± 0.11 ^a^
*GSTR*	1.02 ± 0.19 ^b^	0.75 ± 0.15 ^a^	0.91 ± 0.14 ^ab^	1.27 ± 0.16 ^c^	1.06 ± 0.19 ^b^	0.86 ± 0.14 ^ab^	0.78 ± 0.11 ^a^
*GR*	1.03 ± 0.24 ^b^	0.73 ± 0.11 ^a^	0.95 ± 0.11 ^ab^	1.29 ± 0.25 ^cd^	1.42 ± 0.27 ^d^	1.08 ± 0.14 ^bc^	0.88 ± 0.16 ^ab^
*Nrf2*	1.01 ± 0.16 ^b^	0.64 ± 0.11 ^a^	1.13 ± 0.16 ^bc^	1.27 ± 0.21 ^c^	1.04 ± 0.16 ^b^	0.81 ± 0.12 ^a^	0.77 ± 0.09 ^a^
*Keap1a*	1.01 ± 0.16 ^ab^	1.29 ± 0.25 ^c^	1.11 ± 0.19 ^bc^	0.85 ± 0.17 ^a^	1.11 ± 0.18 ^bc^	1.16 ± 0.22 ^bc^	1.23 ± 0.20 ^bc^
*Keap1b*	1.01 ± 0.15 ^ab^	1.27 ± 0.17 ^c^	0.96 ± 0.14 ^a^	0.89 ± 0.11 ^a^	1.17 ± 0.15 ^bc^	1.21 ± 0.21 ^c^	1.23 ± 0.16 ^c^

Values are means and standard deviations (*n* = 6). ^a, b, c, d^ within a row, means without a common lowercase superscript differ (*p* < 0.05). CuZnSOD, copper/zinc superoxide dismutase; MnSOD, manganese superoxide dismutase; CAT, catalase; GPx, glutathione peroxidase; GST, glutathione-S-transferase; GR, glutathione reductase; Nrf2, NF-E2-related factor 2; Keap1, Kelch-like-ECH-associated protein 1.

**Table 7:** The optimal levels of Met-Met supplementation for juvenile grass carp based on the intestinal antioxidant parameters.

	Parameters	Regression Equation	R^2^	*p*	Optimal Level of Met-Met (g/kg)
PI	MDA	y = 4.0114x^2^ − 13.377x + 29.711	0.9158	0.02	1.67
	PC	y = 0.3073x^2^ − 1.0392x + 2.7039	0.9172	0.03	1.69
	ROS	y = 19.303x^2^ − 70.801x + 160.04	0.9382	0.02	1.83
	AHR	y = −3.5705x^2^ + 11.776x + 34.574	0.8519	0.06	1.65
	ASA	y = −13.701x^2^ + 52.013x + 71.432	0.8589	0.05	1.90
	T-AOC	y = −0.2453x^2^ + 0.8498x + 1.1529	0.8986	0.03	1.73
MI	MDA	y = 3.3909x^2^ − 11.155x + 24.489	0.8824	0.04	1.64
	PC	y = 0.2155x^2^ − 0.7092x + 2.252	0.8987	0.03	1.65
	ROS	y = 19.117x^2^ − 55.073x + 137.57	0.8445	0.06	1.44
	AHR	y = −4.1452x^2^ + 13.877x + 36.22	0.9220	0.02	1.67
	ASA	y = −24.882x^2^ + 81.343x + 107.02	0.9583	0.01	1.67
	T-AOC	y = −0.3335x^2^ + 1.0892x + 1.3004	0.9701	0.01	1.63
DI	MDA	y = 3.4159x^2^ − 11.569x + 27.058	0.9293	0.02	1.69
	PC	y = 0.156x^2^ − 0.5145x + 2.3605	0.8855	0.04	1.65
	ROS	y = 25.517x^2^ − 75.992x + 153.41	0.8690	0.05	1.49
	AHR	y = −4.4182x^2^ + 14.862x + 36.781	0.9305	0.02	1.68
	ASA	y = −16.465x^2^ + 52.281x + 73.494	0.8724	0.05	1.59
	T-AOC	y = −0.2413x^2^ + 0.7319x + 1.5154	0.8167	0.08	1.52

## Data Availability

Data is contained within the article.

## References

[1] Belghit I., Skiba-Cassy S., Geurden I., Dias K., Surget A., Kaushik S., Panserat S., Seiliez I. (2014). Dietary methionine availability affects the main factors involved in muscle protein turnover in rainbow trout (*Oncorhynchus mykiss*). Br. J. Nutr..

[2] Acar Ü., Parrino V., Kesbiç O.S., Lo Paro G., Saoca C., Abbate F., Yılmaz S., Fazio F. (2018). Effects of Different Levels of Pomegranate Seed Oil on Some Blood Parameters and Disease Resistance Against *Yersinia ruckeri* in Rainbow Trout. Front. Physiol..

[3] Parrino V., Kesbic O.S., Acar U., Fazio F. (2020). Hot pepper (*Capsicum* sp.) oil and its effects on growth performance and blood parameters in rainbow trout (*Oncorhynchus mykiss*). Nat. Prod. Res..

[4] Zargar A., Rahimi Afzal Z., Soltani E., Taheri Mirghaed A., Ebrahimzadeh Mousavi H.A., Soltani M., Yuosefi P. (2019). Growth performance, immune response and disease resistance of rainbow trout (*Oncorhynchus mykiss*) fed *Thymus vulgaris* essential oils. Aquac. Res..

[5] He Y., Chi S., Tan B., Dong X., Yang Q., Liu H., Zhang S., Han F., Liu D. (2019). DL-Methionine supplementation in a low-fishmeal diet affects the TOR/S6K pathway by stimulating ASCT2 amino acid transporter and insulin-like growth factor-I in the dorsal muscle of juvenile cobia (*Rachycentron canadum*). Br. J. Nutr..

[6] Li X., Zheng S., Wu G., Wu G. (2021). Nutrition and Functions of Amino Acids in Fish. Amino Acids in Nutrition and Health.

[7] Wassef E.A., Saleh N.E., Ashry A.M. (2021). Taurine or Sodium Diformate Supplementation to a Low Fishmeal Plant-Based Diet Enhanced Immunity and Muscle Cellularity of European Sea-Bass (*Dicentrarchus labrax*). J. FisheriesSciences.com.

[8] Aoki H., Akimoto A., Watanabe T. (2001). Periodical changes of plasma free amino acid levels and feed digesta in yellowtail after feeding non-fishmeal diets with or without supplemental crystalline amino acids. Fish. Sci..

[9] Zhou X.Q., Zhao C.R., Lin Y. (2007). Compare the effect of diet supplementation with uncoated or coated lysine on juvenile Jian Carp (*Cyprinus carpio* Var. Jian). Aquac. Nutr..

[10] Yuan Y., Gong S., Yang H., Lin Y., Yu D., Luo Z. (2011). Effects of supplementation of crystalline or coated lysine and/or methionine on growth performance and feed utilization of the Chinese sucker, *Myxocyprinus asiaticus*. Aquaculture.

[11] Parker S.F., Funnell N.P., Shankland K., Kabova E.A., Häußner T., Hasselbach H., Braune S., Kobler C., Albers P.W. (2021). Structure and spectroscopy of methionyl-methionine for aquaculture. Sci. Rep..

[12] Su Y., Wu P., Feng L., Jiang W., Jiang J., Zhang Y., Figueiredo-Silva C., Zhou X., Liu Y. (2018). The improved growth performance and enhanced immune function by DL methionyl-DL-methionine are associated with NF-κB and TOR signalling in intestine of juvenile grass carp (*Ctenopharyngodon idella*). Fish Shellfish Immunol..

[13] Guo T., Zhao W., He J., Liao S., Xie J., Xie S., Masagounder K., Liu Y., Tian L., Niu J. (2020). Dietary DL-methionyl-DL-methionine supplementation increased growth performance, antioxidant ability, the content of essential amino acids and improved the diversity of intestinal microbiota in Nile tilapia (*Oreochromis niloticus*). Br. J. Nutr..

[14] Xie J.J., Lemme A., He J.Y., Yin P., Figueiredo-Silva C., Liu Y.J., Xie S.W., Niu J., Tian L.X. (2018). Fishmeal levels can be successfully reduced in white shrimp (*Litopenaeus vannamei*) if supplemented with DL-Methionine (DL-Met) or DL-Methionyl-DL-Methionine (Met-Met). Aquac. Nutr..

[15] Ji R., Wang Z., He J., Masagounder K., Xu W., Mai K., Ai Q. (2021). Effects of DL-methionyl-DL-methionine supplementation on growth performance, immune and antioxidative responses of white leg shrimp (*Litopenaeus vannamei*) fed low fishmeal diet. Aquac. Rep..

[16] Niklasson L., Sundh H., Fridell F., Taranger G.L., Sundell K. (2011). Disturbance of the intestinal mucosal immune system of farmed Atlantic salmon (*Salmo salar*), in response to long-term hypoxic conditions. Fish Shellfish Immunol..

[17] Pijls K.E., Jonkers D.M., Elamin E.E., Masclee A.A., Koek G.H. (2013). Intestinal epithelial barrier function in liver cirrhosis: An extensive review of the literature. Liver Int..

[18] Yang J., Wang C., Xu Q., Zhao F., Liu J., Liu H. (2015). Methionyl-Methionine Promotes α-s1 Casein Synthesis in Bovine Mammary Gland Explants by Enhancing Intracellular SubstrateAvailability and Activating JAK2-STAT5 and mTOR-Mediated Signaling Pathways. J. Nutr..

[19] Sakai I., Kraft A.S. (1997). The Kinase Domain of Jak2 Mediates Induction of Bcl-2 and Delays Cell Death in Hematopoietic Cells. J. Biol. Chem..

[20] Zhong C., Tong D., Zhang Y., Wang X., Yan H., Tan H., Gao C. (2022). DL-methionine and DL-methionyl-DL-methionine increase intestinal development and activate Wnt/b-catenin signaling activity in domestic pigeons (*Columba livia*). Poult. Sci..

[21] Pan F., Feng L., Jiang W., Jiang J., Wu P., Kuang S., Tang L., Tang W., Zhang Y., Zhou X. (2016). Methionine hydroxy analogue enhanced fish immunity via modulation of NF-κB, TOR, MLCK, MAPKs and Nrf2 signaling in young grass carp (*Ctenopharyngodon idella*). Fish Shellfish Immunol..

[22] Zeng Y.Y., Jiang W.D., Liu Y., Wu P., Zhao J., Jiang J., Kuang S.Y., Tang L., Tang W.N., Zhang Y.A. (2016). Optimal dietary alpha-linolenic acid/linoleic acid ratio improved digestive and absorptive capacities and target of rapamycin gene expression of juvenile grass carp (*Ctenopharyngodon idellus*). Aquac. Nutr..

[23] NRC (2011). Nutrient Requirements of Fish and Shrimp.

[24] Hsu C., Chiu Y. (2009). Ambient temperature influences aging in an annual fish (*Nothobranchius rachovii*). Aging Cell.

[25] Martínez-Álvarez R.M., Morales A.E., Sanz A. (2005). Antioxidant defenses in fish: Biotic and abiotic factors. Rev. Fish Biol. Fish..

[26] Lu J., Zhang X., Zhou Q., Cheng Y., Luo J., Masagounder K., He S., Zhu T., Yuan Y., Shi B. (2021). Dietary DL-methionyl-DL-methionine supplementation could improve growth performance under low fishmeal strategies by modulating TOR signalling pathway of *Litopenaeus vannamei*. Aquac. Nutr..

[27] Tiedge M., Lortz S., Drinkgern J., Lenzen S. (1997). Relation between antioxidant enzyme gene expression and antioxidative defense status of insulin-producing cells. Diabetes.

[28] Casetta J., Ribeiro R.P., Lewandowski V., Khatlab A.S., de Oliveira N.A., Boscolo W.R., Gasparino E. (2021). Expression of the PEPT1, CAT, SOD2 and GPX1 genes in the zebrafish intestine supplemented with methionine dipeptide under predation risk. J. Anim. Physiol. Anim. Nutr..

[29] Zelko I.N., Mariani T.J., Folz R.J. (2002). Superoxide dismutase multigene family: A comparison of the CuZn-SOD (SOD1), Mn-SOD (SOD2), and EC-SOD (SOD3) gene structures, evolution, and expression. Free Radic. Biol. Med..

[30] Bender A., Hajieva P., Moosmann B. (2008). Adaptive Antioxidant Methionine Accumulation in Respiratory Chain Complexes Explains the Use of a Deviant Genetic Code in Mitochondria. Proc. Natl. Acad. Sci. USA.

[31] Taguchi K., Maher J.M., Suzuki T., Kawatani Y., Motohashi H., Yamamoto M. (2010). Genetic analysis of cytoprotective functions supported by graded expression of Keap1. Mol. Cell. Biol..

[32] Shay K.P., Michels A.J., Li W., Kong A.N., Hagen T.M. (2012). Cap-independent Nrf2 translation is part of a lipoic acid-stimulated detoxification stress response. Biochim. Biophys. Acta.

[33] Bruewer M., Luegering A., Kucharzik T., Parkos C.A., Madara J.L., Hopkins A.M., Nusrat A. (2003). Proinflammatory cytokines disrupt epithelial barrier function by apoptosis-independent mechanisms. J. Immunol..

[34] Elmore S. (2007). Apoptosis: A Review of Programmed Cell Death. Toxicol. Pathol..

[35] Chasiotis H., Kelly S.P. (2011). Effect of cortisol on permeability and tight junction protein transcript abundance in primary cultured gill epithelia from stenohaline goldfish and euryhaline trout. Gen. Comp. Endocrinol..

[36] Chasiotis H., Kolosov D., Bui P., Kelly S.P. (2012). Tight junctions, tight junction proteins and paracellular permeability across the gill epithelium of fishes: A review. Respir. Physiol. Neurobiol..

[37] Weber C.R., Raleigh D.R., Su L., Shen L., Sullivan E.A., Wang Y., Turner J.R. (2010). Epithelial myosin light chain kinase activation induces mucosal interleukin-13 expression to alter tight junction ion selectivity. J. Biol. Chem..

[38] Ito T., Saitoh D., Takasu A., Kiyozumi T., Sakamoto T., Okada Y. (2004). Serum cortisol as a predictive marker of the outcome in patients resuscitated after cardiopulmonary arrest. Resuscitation.

[39] Lis M.T., Crampton R.F., Matthews D.M. (1972). Effect of dietary changes on intestinal absorption of L-methionine and L-methionyl-L-methionine in the rat. Br. J. Nutr..

[40] Pan Y., Bender P.K., Akers R.M., Webb K.J. (1996). Methionine-containing peptides can be used as methionine sources for protein accretion in cultured C2C12 and MAC-T cells. J. Nutr..

[41] Jiang W., Deng Y., Liu Y., Qu B., Jiang J., Kuang S., Tang L., Tang W., Wu P., Zhang Y. (2015). Dietary leucine regulates the intestinal immune status, immune-related signalling molecules and tight junction transcript abundance in grass carp (*Ctenopharyngodon idella*). Aquaculture.

[42] Deng Y., Jiang W., Liu Y., Jiang J., Kuang S., Tang L., Wu P., Zhang Y., Feng L., Zhou X. (2014). Differential growth performance, intestinal antioxidant status and relative expression of Nrf2 and its target genes in young grass carp (*Ctenopharyngodon idella*) fed with graded levels of leucine. Aquaculture.

[43] Morales L.E., Higuchi A. (2018). Is fish worth more than meat?—How consumers’ beliefs about health and nutrition affect their willingness to pay more for fish than meat. Food Qual. Prefer..

[44] Baldissera M.D., Souza C.F., Zeppenfeld C.C., Velho M.C., Klein B., Abbad L.B., Ourique A.F., Wagner R., Da Silva A.S., Baldisserotto B. (2020). Dietary supplementation with nerolidol nanospheres improves growth, antioxidant status and fillet fatty acid profiles in Nile tilapia: Benefits of nanotechnology for fish health and meat quality. Aquaculture.

